# Detection of cerebral hypoperfusion with a dynamic hyperoxia test using brain oxygenation pressure monitoring

**DOI:** 10.1186/s13054-022-03918-0

**Published:** 2022-02-07

**Authors:** Thomas Gargadennec, Gioconda Ferraro, Rudy Chapusette, Xavier Chapalain, Elisa Bogossian, Morgane Van Wettere, Lorenzo Peluso, Jacques Creteur, Olivier Huet, Niloufar Sadeghi, Fabio Silvio Taccone

**Affiliations:** 1 Department of Intensive Care, Hospital Erasme, Université Libre de Bruxelles (ULB), Route de Lennik, 808, 1070 Brussels, Belgium; 2 Département d’Anesthésie et Réanimation Chirurgicale, Centre Hospitalier Universitaire de Brest, Université de Bretagne Occidentale (UBO), Brest, France; 3 Department of Radiology, Hospital Erasme, Université Libre de Bruxelles (ULB), Brussels, Belgium

**Keywords:** Brain injury, Oxygen test, Hypoperfusion, Multimodal monitoring

## Abstract

**Introduction:**

Brain multimodal monitoring including intracranial pressure (ICP) and brain tissue oxygen pressure (PbtO_2_) is more accurate than ICP alone in detecting cerebral hypoperfusion after traumatic brain injury (TBI). No data are available for the predictive role of a dynamic hyperoxia test in brain-injured patients from diverse etiology.

**Aim:**

To examine the accuracy of ICP, PbtO_2_ and the oxygen ratio (OxR) in detecting regional cerebral hypoperfusion, assessed using perfusion cerebral computed tomography (CTP) in patients with acute brain injury.

**Methods:**

Single-center study including patients with TBI, subarachnoid hemorrhage (SAH) and intracranial hemorrhage (ICH) undergoing cerebral blood flow (CBF) measurements using CTP, concomitantly to ICP and PbtO_2_ monitoring. Before CTP, FiO_2_ was increased directly from baseline to 100% for a period of 20 min under stable conditions to test the PbtO_2_ catheter, as a standard of care. Cerebral monitoring data were recorded and samples were taken, allowing the measurement of arterial oxygen pressure (PaO_2_) and PbtO_2_ at FiO_2_ 100% as well as calculation of OxR (= ΔPbtO_2_/ΔPaO_2_). Regional CBF (rCBF) was measured using CTP in the tissue area around intracranial monitoring by an independent radiologist, who was blind to the PbtO_2_ values. The accuracy of different monitoring tools to predict cerebral hypoperfusion (i.e., CBF < 35 mL/100 g × min) was assessed using area under the receiver-operating characteristic curves (AUCs).

**Results:**

Eighty-seven CTPs were performed in 53 patients (median age 52 [41–63] years—TBI, *n* = 17; SAH, *n* = 29; ICH, *n* = 7). Cerebral hypoperfusion was observed in 56 (64%) CTPs: ICP, PbtO_2_ and OxR were significantly different between CTP with and without hypoperfusion. Also, rCBF was correlated with ICP (*r* = − 0.27; *p* = 0.01), PbtO_2_ (*r* = 0.36; *p* < 0.01) and OxR (*r* = 0.57; *p* < 0.01). Compared with ICP alone (AUC = 0.65 [95% CI, 0.53–0.76]), monitoring ICP + PbO_2_ (AUC = 0.78 [0.68–0.87]) or ICP + PbtO_2_ + OxR (AUC = 0.80 (0.70–0.91) was significantly more accurate in predicting cerebral hypoperfusion. The accuracy was not significantly different among different etiologies of brain injury.

**Conclusions:**

The combination of ICP and PbtO_2_ monitoring provides a better detection of cerebral hypoperfusion than ICP alone in patients with acute brain injury. The use of dynamic hyperoxia test could not significantly increase the diagnostic accuracy.

**Supplementary Information:**

The online version contains supplementary material available at 10.1186/s13054-022-03918-0.

## Introduction

Acute severe brain injury, either secondary to trauma or non-traumatic events, is still associated with a significant burden of long-term neurological sequelae [1, 2] and represents one of the major causes of morbidity and mortality in previously healthy population [3]. On the opposite to the initial brain injury, whose severity can be barely modified, secondary brain injuries (SBIs) could be detected and potentially avoidable. Those SBI, including either cerebral (i.e., brain edema, tissue hypoxia, seizures) or systemic (i.e., hypotension, hypocapnia, hypoxemia, dysglycemia, hyponatremia, fever and anemia) events, can enhance the extent of the primary brain insult and further contribute to poor outcome in this setting [4–7].

Following the occurrence of SBI, cerebral blood flow (CBF) might become inadequate to provide sufficient oxygen and nutrients supply to meet the metabolic needs of the injured brain [8]. Imaging techniques can provide relevant information on CBF alterations after acute brain injury; in particular, cerebral computed tomography perfusion (CTP) imaging, which was initially introduced to estimate the infarct core size and evaluate the time window for thrombolysis and thrombectomy in ischemic stroke [9], can detect perfusion deficits associated with cerebral vasospasm, which might occur in patients suffering from subarachnoid hemorrhage (SAH), or reduced CBF around contusion areas and cerebral perfusion heterogeneity in the early phase of TBI [10, 11]. However, CTP is not a bedside tool, does not provide continuous CBF measurement and can be associated with some adverse events, such as high-dose radiation exposure and an increased risk of elevated intracranial pressure (ICP) during the in-hospital transfer to the radiology unit [12].

As such, alternative monitoring tools are available to estimate the occurrence of brain hypoperfusion at the bedside. Among them, ICP and cerebral perfusion pressure (CPP) monitoring are widely recommended, in particular for TBI patients, to identify patients at risk of brain hypoperfusion. Nevertheless, CBF might be inadequate even in the absence of abnormal ICP/CPP values [13]; as such, invasive brain tissue oxygenation (PbtO_2_) monitoring could provide additional information on the equilibrium between oxygen delivery, diffusion and consumption within the brain parenchyma and detect the occurrence of tissue hypoxia even in the absence of elevated ICP [14]. In one study, the combination of ICP and PbtO_2_ monitoring was more accurate than ICP monitoring alone in detecting cerebral hypoperfusion in TBI patients [15]. However, this study included only 30 CTPs and focused only on TBI with predominantly diffuse injury, which would limit the generalizability of these findings to a larger cohort of brain-injured patients. Moreover, PbtO_2_ cannot directly reflect CBF values, as brain oxygenation is also influenced by other factors, such as hemoglobin values, brain temperature, microvascular impairment and arterial oxygenation [16]. Changes in arterial oxygen pressure (PaO_2_) could result in concomitant changes in PbtO_2_, whose magnitude is dependent from local regulatory mechanisms, brain metabolism as well as the adequacy of regional perfusion. As such, one may argue that a challenge of increased arterial oxygenation could provide a more significant increase in PbtO_2_ in those area with sufficient vascular supply, while the tissue oxygen response would be more limited in hypoperfused areas. In one study, van Santibrink et al. showed that increased tissue oxygen response was associated with poor prognosis in TBI patients [17]; however, no data on the association of such response with regional perfusion were provided.

Therefore, the aim of this study was to assess the role of a dynamic oxygen challenge to identify cerebral hypoperfusion in brain-injured patients. We hypothesized that a higher tissue oxygen response could correlate with increased regional CBF values in these patients.

## Methods

### Study population

This was an analysis of prospectively collected data including all adult (> 18 years of age) patients with an acute primary brain injury (i.e., TBI, SAH or intracranial hemorrhage, ICH) admitted to the ICU of Erasme Hospital, Brussels, Belgium, between January 2016 and August 2019. Eligible patients were those: (a) having a PbtO_2_ monitoring catheter, which was inserted according to the decision of senior ICU physician and an experienced neurosurgeon; (b) underwent daily dynamic oxygen challenge (see below) as part of routine assessment of PbtO_2_ function; (c) underwent a CTP within 3 h from the dynamic oxygen challenge. Data for all measurements were recorded into the patient management data system (PDMS, Picis Critical Care Manager, Picis Inc., Wakefield, USA). Exclusion criteria were a malfunctioning PbtO_2_ catheter, the lack of data on the dynamic oxygen challenge, poor-quality CTP images (i.e., inadequate contrast medium injection and/or artifacts), baseline-inspired oxygen fraction (FiO_2_) on the ventilator ≥ 80% and the use of prone positioning and/or extra-corporeal membrane oxygenation. The study was approved by the ethical committee of the Erasme Hospital (Comité d’Ethique Hospitalo—Facultaire Erasme—ULB; P2021/348), which waived the need of informed consent given the observational design of the study analyzing recorded data into the PDMS.

### Patients’ management and dynamic oxygen challenge

Patients were managed according to local protocols, based on international recommendations [18–20]. A triple lumen bolt allowing the insertion of a PbtO_2_ probe (IM3.ST_EU, Integra LifeSciences Corporation, Plainsboro, NJ, USA), alone or in association with an 8-contact depth EEG electrode and a microdialysis catheter, was placed in the operating room by a neurosurgeon in patients with TBI, SAH or ICH, who had indications for ICP monitoring (i.e., abnormal CT-scan findings and a Glasgow Coma Score on admission < 9). The bolt was positioned in the normal-appearing brain area of the injured side (TBI or ICH) or, in case of aneurysmal SAH, on either the ipsilateral side of the aneurysm (i.e., anterior circulation) or on the right side (i.e., no aneurysm identified or aneurysm located in the posterior circulation). Other continuously monitored variables included heart rate, mean arterial pressure (MAP), peripheral oxygen saturation, end-tidal carbon dioxide and body temperature (i.e., with urinary or esophageal probes), ICP and CPP; CPP was calculated as the difference between MAP and ICP; MAP was zeroed at the level of the left atrium. ICP and CPP targets were < 20 and > 60 mmHg, respectively.

After the initial daily assessment of the patient including arterial blood gas analyses (ABG), a dynamic oxygen challenge, i.e., increasing of FiO_2_ to 100% for 20 min, was performed and another ABG repeated at the end of this period. As such, PaO_2_ and PbtO_2_ were measured at baseline and after the dynamic oxygen challenge. This dynamic challenge was part of the routine management of patients and performed by an experienced intensivist (FST), whenever possible. Apart from the dynamic oxygen challenge, all other relevant physiological variables were kept stable. The oxygen ratio (OxR) was then calculated as follows: OxR = (PbtO_2_ at FiO_2_ 100% − PbtO_2_ at baseline)/(PaO_2_ at FiO_2_ 100% − PaO_2_ at baseline).

### Data collection

For all patients, demographics, comorbid diseases, reasons for ICU admission as well as ICU length of stay and hospital mortality were collected. The severity of disease scores (i.e., Glasgow Coma Scale on admission, World Federation of Neurological Surgeons—WFNS—score in SAH patients, Marshall and modified Fisher scores for cerebral CT-scan in TBI or SAH patients, respectively, location and volume of ICH) was collected. Intracranial hypertension was defined by the ICP value above 20 mmHg immediately before the dynamic challenge test; also, brain tissue hypoxia was defined by a PbtO_2_ below 20 mmHg at the same moment.

Neurological outcome at hospital discharge was assessed using the Glasgow Outcome Scale (GOS); favorable neurological outcome (FO) was considered as a GOS 4–5, while unfavorable outcome (UO) as GOS 1–3.

### Cerebral CT perfusion scan

**Fig. 1:**
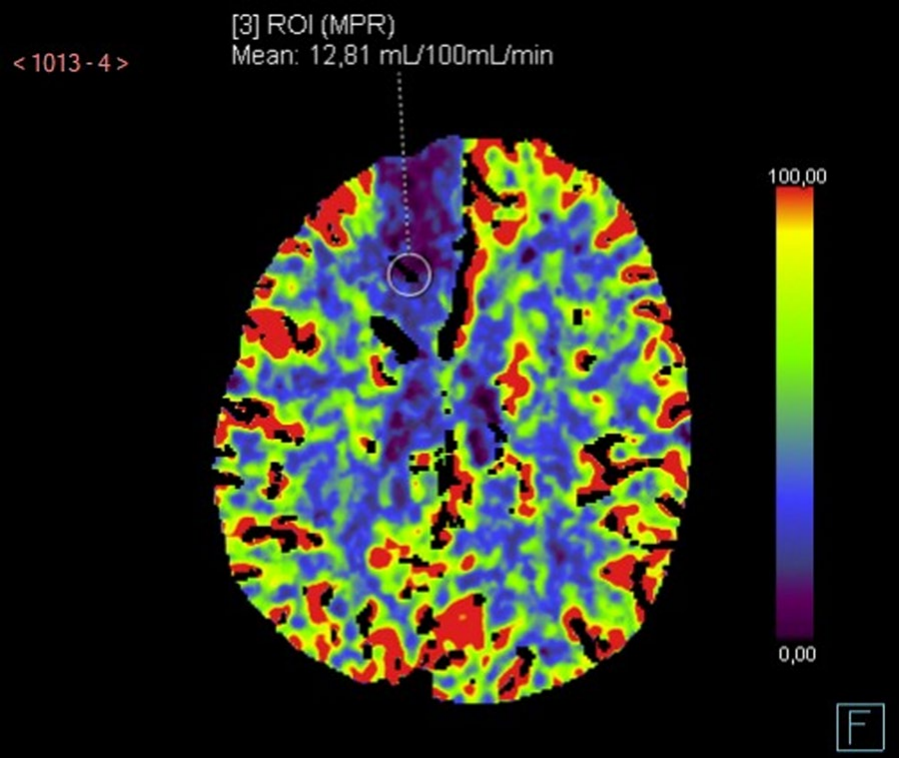
Patient with severe subarachnoid hemorrhage, who underwent a cerebral CT-perfusion (CTP) on day 2. White circle indicated the region of interest (ROI) for CTP analysis of regional cerebral blood flow (rCBF); rCBF was estimated at 12.8 mL/100 g × min, while intracranial pressure and cerebral perfusion pressure were 16 mmHg and 73 mmHg, respectively, and baseline PbtO_2_ was 22 mmHg (for a PaO_2_ of 119 mmHg). Measured OxR was 0.14

Cerebral CTP was performed using a multidetector row CT (FORCE Dual Energy, Siemens Healthcare, Erlangen, Germany) and considered the reference method to identify areas of cerebral hypoperfusion. The indication for CTP was based on the decision of the attending ICU physician after discussion with a senior radiologist and, in general, based on the suspicion of cerebral vasospasm (SAH) or to assess the extension of cerebral hypoperfusion (TBI, ICH); the decision to perform CTP was independent from the results of the dynamic hyperoxia test. All available CTPs paired by a dynamic oxygen challenge were considered for the final analysis. The method of acquisition was similar to what previously described by Bouzat et al. [15]; scanning was initiated 5 s after injection of 50 mL of iohexol (300 mg/mL of iodine; GE Healthcare, Milwaukee, WI), at a rate of 5 mL/s, with the following variables: 80 kV, 240 mAs, 0.4 rotations/s, and total duration of 50 s. The series evaluated 16 adjacent 5-mm-thick sections of brain parenchyma. Post-processing of CTP data was performed by two experienced neuroradiologists, using a dedicated software (Brilliance Workspace Portal; Philips Medical Systems, Cleveland, OH), focusing on one region of interest (ROI), which was manually drawn around the probe (surface area, ~ 50 mm^2^) to calculate regional CBF (Fig. 1). Assessment of regional CBF was performed blindly to ICP and PbtO_2_ data. Low regional cerebral blood flow (rCBF) was defined by a value below 35 mL/100 g × min [15].

### Study outcomes

The primary outcome of the study was to compare the accuracy of ICP, PbtO_2_ or OxR and their combinations to detect cerebral hypoperfusion in brain-injured patients. Secondary outcomes included: (a) the accuracy to detect cerebral hypoperfusion of all these variables according to the underlying brain disease.

### Statistical analysis

Data were analyzed using R statistical software version 4.0.3 (R Foundation for Statistical Computing), Prism (GraphPad Software Inc.) and IBM SPSS Statistics for Macintosh 27 (Armonk, NY, USA). Categorical variables were expressed as count (percentage) and continuous variables as mean ± standard deviation (SD) or median [25th–75th percentiles]. The Kolmogorov–Smirnov test was used, and histograms and normal-quantile plots were examined to verify the normality of distribution of continuous variables. Differences between groups were assessed using the Chi-square test or Fisher’s exact test for categorical variables and Student’s t-test, or Mann–Whitney U-test for continuous variables, as appropriate. The discriminative ability of each variable or combination to predict cerebral hypoperfusion was evaluated using receiver operating characteristic (ROC) curves with the corresponding area under the curve (AUROC), and sensitivity, specificity, positive (PPV) and negative predictive value (NPV) were computed. For each variable, the optimal predictive threshold was calculated using the Youden’s index. Differences between AUROCs were assessed using the DeLong analysis. Correlations between monitoring variables and CTP data were measured with Pearson’s correlation coefficient. All tests were two-tailed, and statistical significance was set at the 5% level.

## Results

### Study population

Over the study period, 123 patients underwent PbtO_2_ monitoring; of those, 70 (*n* = 43, no CTP; *n* = 4, poor-quality imaging; *n* = 9 with FiO_2_ > 80%; *n* = 14, no dynamic challenge test—Additional file 1: Fig. S1) were excluded leaving 53 patients for the final analysis. Characteristics of the study population are shown in Table 1; median age was 52 [41–63] years; and 25 (44%) were female. Admission diagnosis was SAH in 29 (51%), TBI in 17 (30%) and ICH in 7 (12%) patients. Overall mortality was 28%; median GOS at hospital discharge was 3 [1–3].

**Table 1:** Characteristics of the study population. Data are presented as count (%) or median [IQRs]

	*n* = 53
Age, years	52 [41–63]
Female/male ratio	25/28
GCS on admission	9 [5–14]
Subarachnoid hemorrhage	29
*Fisher classification*	
3	7
4	22
*WFNS classification*	
1	5
2	4
3	0
4	7
5	13
Traumatic brain injury	17
*Marshall classification*	
3	4
4	6
5	2
6	5
Intracerebral hemorrhage	7
*Hematoma > 30 mL*	6
GOS at hospital discharge	3 [1–3]
30-day mortality	15 (28)
ICU length of stay, days	21 [15–29]

*GCS* Glasgow Coma Scale, *WFNS* World Federation of Neurosurgical Societies, *GOS* Glasgow Outcome Scale, *ICU* intensive care unit

### Cerebral CT perfusion

A total of 87 CTPs were performed; one patient had 6 examinations, two underwent 4 CTPs, one patient 3 CTPs, twenty-one 2 CTPs and the 28 remaining patients one CTP; 56 (64%) CTPs were performed in SAH, 20 (23%) in TBI and 11 (13%) in ICH patients. The median time between the insertion of PbtO_2_ catheter and CTP was 6 [3–9] days; no correlation between the time to insertion of the catheter and baseline PbtO_2_ (*r* = 0.001; *p* = 0.97) and PbtO_2_ at FiO_2_ of 100% (*r* = − 0.13; *p* = 0.24) was observed.

### Brain hypoperfusion and dynamic oxygen test

**Fig. 2:**
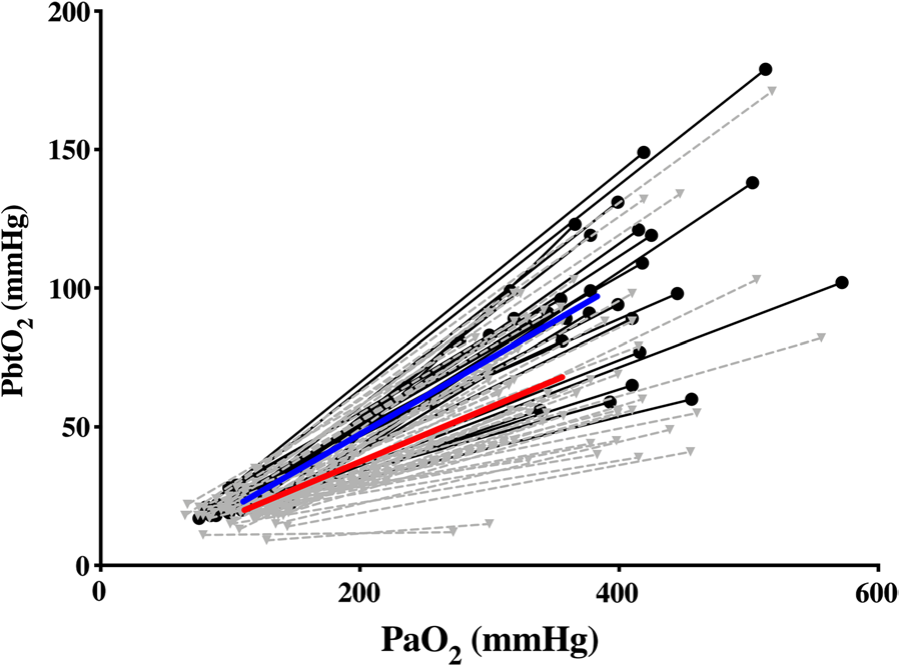
Representation of brain tissue oxygen pressure (PbtO_2_) and arterial blood partial pressure of oxygen (PaO_2_) during hyperoxia test at FiO_2_ 100%. Each test is represented by two points united by one straight line. Depending on regional cerebral blood flow (rCBF) points and lines are black full circles united by black lines (normal rCBF) or grey triangles united by grey dotted lines (oligemia, i.e., regional cerebral blood flow < 35 mL/100 g × min). The blue line unit means of PbtO_2_ and PbtO_2_ at FiO_2_ 100% in the group with normal rCBF and the red line units means in the group with oligemia

Fifty-six (64%) CTPs were associated with regional cerebral hypoperfusion; CTPs showing cerebral hypoperfusion had higher ICP, lower PbtO_2_ at baseline and at FiO_2_ of 100% and lower OxR (Fig. 2) when compared to others; however, no differences in CPP, PaO_2_, hemoglobin and PaCO_2_ were observed between the two groups (Table 2).

**Table 2:** Differences in main available data on the day of cerebral CT perfusion (CTP), according to the presence of brain hypoperfusion (i.e., regional cerebral blood flow < 35 mL/100 g × min). Data are presented as count (%) or median [IQRs]

	ALL (*n* = 87)	Brain hypoperfusion (*n* = 56)	No brain hypoperfusion (*n* = 31)	*p* value
*Baseline*				
ICP, mmHg	13 [9–17]	15 [9–19]	11 [8–15]	0.02
CPP, mmHg	94 [80–110]	95 [81–112]	86 [79–108]	0.36
PbtO_2_, mmHg	21 [19–23]	20 [18–22]	22 [21–25]	< 0.01
PaO_2_, mmHg	110 [98–124]	112 [97–127]	109 [99–122]	0.79
FiO_2_ at baseline, %	35 [30–40]	35 [30–40]	30 [30–40]	0.04
PaO_2_/FiO_2_ at baseline	327 [283–365]	312 [269–357]	338 [297–370]	0.10
PEEP, cmH_2_O	8 [5–10]	8 [5–10]	8 [5–10]	0.68
pH	7.42 [7.38–7.44]	7.42 [7.38–7.44]	7.41 [7.39–7.43]	0.82
PaCO_2_ baseline, mmHg	39 [36–43]	39 [37–42]	38 [35–41]	0.06
Sodium, mmol/L	140 [138–144]	140 [138–144]	140 [138–142]	0.59
Hemoglobin, g/dL	10.3 [9.1–11.4]	10.2 [9.6–11.6]	10.5 [8.9–11.3]	0.56
Glucose, mg/dL	134 [121–145]	133 [119–144]	138 [126–154]	0.20
Body temperature, °C	37.1 [36.8–37.5]	37.2 [36.7–37.6]	37.1 [36.9–37.5]	0.98
Sedatives, n (%)	48 (55)	31 (55)	17 (55)	1.00
Opioids, n (%)	54 (62)	34 (61)	20 (65)	0.82
NMBAs, n (%)	22 (25)	14 (25)	8 (26)	1.00
Norepinephrine, n (%)	66 (76)	43 (77)	23 (74)	0.80
Inotropic agents, n (%)	20 (23)	13 (23)	7 (23)	1.00
*End of the dynamic oxygen challenge*				
PbtO_2_, mmHg	79 [56–95]	62 [46–88]	91 [81–119]	< 0.01
PaO_2_, mmHg	359 [319–410]	345 [315–407]	378 [339–418]	0.08
pH	7.42 [7.38–7.43]	7.42 [7.37–7.44]	7.42 [7.39–7.43]	0.74
PaCO_2_, mmHg	39 [36–44]	39 [36–44]	38 [36–42]	0.72
Oxygen ratio	0.23 [0.15–0.29]	0.21 [0.12–0.27]	0.28 [0.22–0.33]	< 0.01
rCBF, mL/100 g × min	31.3 [22.6–41.3]	25.6 [15.4–31.2]	49.3 [40.4–68.0]	< 0.01

*ICP* Intracranial pressure, *CPP* cerebral perfusion pressure, *PbtO*_*2*_ brain tissue oxygen pressure, *PaO*_*2*_ arterial blood partial pressure of oxygen, *rCBF* regional cerebral blood flow, *NMBA* neuromuscular blocking agents

**Fig. 3:**
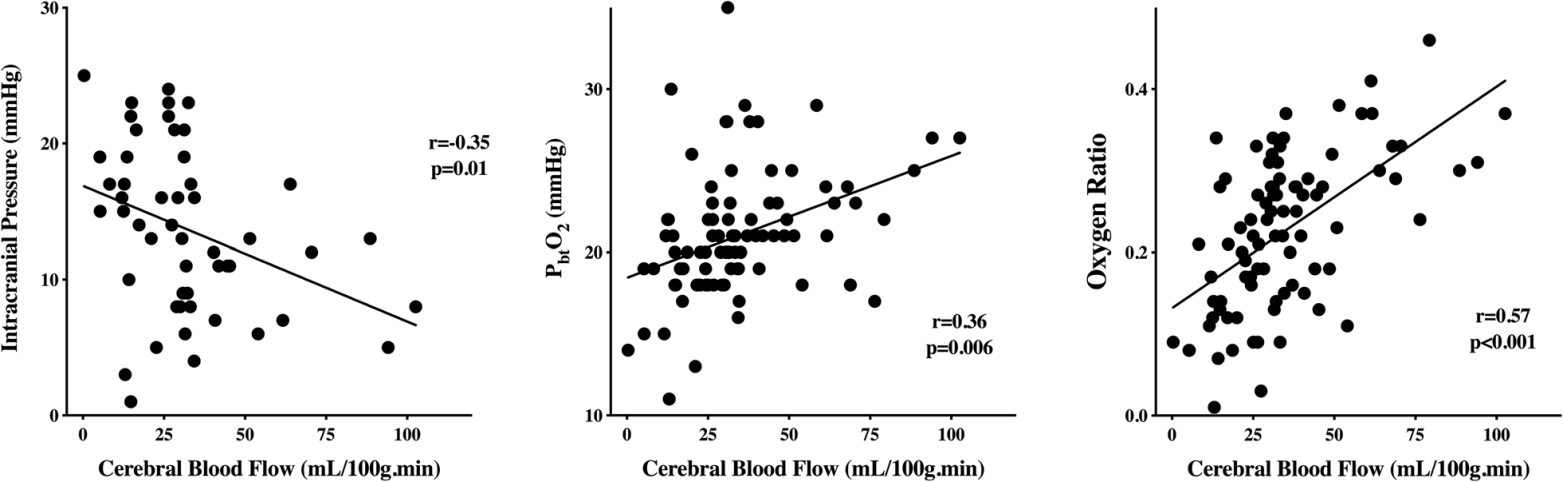
Correlation between baseline intracranial pressure, baseline brain oxygen pressure (PbtO_2_) and oxygen ratio with regional cerebral blood flow (CBF)

The OxR had the highest correlation with regional CBF (*r* = 0.57; *p* < 0.001), when compared to ICP (*r* = − 0.35; *p* = 0.01) and PbtO_2_ at baseline (*r* = 0.36; *p* = 0.006—Fig. 3). An ICP > 20 mmHg had a specificity of 21% and a sensitivity of 97% to detect brain hypoperfusion, while a PbtO_2_ < 20 mmHg had a specificity of 62% and a sensitivity of 84% to detect brain hypoperfusion. An OxR < 0.20 had a 48% specificity and 81% sensitivity to detect brain hypoperfusion.

Intracranial hypertension was present in 13 (25%) of CTPs; of those, 12 (92%) had brain hypoperfusion; among the 74 CTPs without intracranial hypertension, 44 (60%) had brain hypoperfusion (Additional file 1: Fig. S2). Brain hypoxia was present in 30 (58%) CTPs; of those, 26 (87%) had brain hypoperfusion; among the 57 CTPs without brain hypoxia, 30 (52%) had brain hypoperfusion. A total of 23/56 (41%) with CTPs showing brain hypoperfusion had neither intracranial hypertension nor tissue hypoxia (Additional file 1: Table S1). Low OxR (i.e.,  < 0.20) was present in 33 (38%) of CTPs; of those, 27 (81%) had brain hypoperfusion; among the 54 CTPs without low OxR, 29 (54%) had brain hypoperfusion. A total of 11 out of 23 CTPs with brain hypoperfusion showed only low OxR, without intracranial hypertension or tissue hypoxia (Additional file 1: Fig. S2). While the combination of at least two abnormal parameters between ICP (> 20 mmHg), PbtO_2_ (< 20 mmHg) and OxR (< 0.20) resulted in brain hypoperfusion on CTP in 82–100% of cases, the absence of at least of these two abnormal values was associated with brain hypoperfusion in 39–47% of cases (Additional file 1: Figs. S2 and S3).

### Predictors of brain hypoperfusion

As reported in Table 3 and Fig. 2, the most accurate variables to detect brain hypoperfusion were PbtO_2_ at baseline and OxR. Different combinations of neuromonitoring data (i.e., ICP with PbtO_2_; ICP with OxR; ICP with PbtO_2_ and OxR) resulted in significantly higher AUC than ICP alone (*p* = 0.01, *p* = 0.04 and *p* = 0.02 vs. ICP, respectively); the combination of ICP and PbtO_2_ yielded similar results than the combination of ICP, PbtO_2_ and OxR.

**Table 3:** Correlation between regional CBF and monitoring parameters. The discriminative ability of each variable or combination to predict cerebral hypoperfusion (i.e., regional CBF < 35 mL/100 g × min) was evaluated using receiver operating characteristic curves with the corresponding area under the curve (AUROC), and sensitivity, specificity, positive (PPV) and negative predictive value (NPV) were computed

	Correlation with rCBF	AUROC (95% CI)
ICP	*r* = − 0.27; *p* = 0.01	0.65 (0.53–0.76)
PbtO_2_	*r* = 0.36; *p* < 0.01	0.75 (0.64–0.85)
Oxygen ratio	*r* = 0.57; *p* < 0.01	0.75 (0.63–0.85)
CPP	*r* = 0.01; *p* = 0.92	0.57 (0.44–0.70)
ICP + PbtO_2_	–	0.78 (0.68–0.87)
ICP + OxR	–	0.78 (0.68–0.88)
ICP + PbO_2_ + oxygen ratio	–	0.80 (0.70–0.91)

*ICP* intracranial pressure, *CPP* cerebral perfusion pressure, *PbtO*_*2*_ brain tissue oxygen pressure, *CI* confidence intervals

### Subgroup analyses

Differences in main characteristics between patients with and without hypoperfusion on CTP, according to the presence of a traumatic (*n* = 19) or non-traumatic (*n* = 68) brain injury, are reported in Additional file 1: Tables S2 and S3. Correlations of ICP, PbtO_2_ and OxR with rCBF are reported, according to the presence of a traumatic or non-traumatic brain injury, in Additional file 1: Tables S4 and S5; results were similar to those of the entire cohort. In CTP associated with TBI (*n* = 19), the highest AUC to predict brain hypoperfusion was observed for the combination of ICP, PbtO_2_ at baseline and OxR (0.90 [0.75–1.00]), although it was not significantly different than ICP and PbtO_2_ or ICP and OxR (Additional file 1: Table S4). In CTP with non-traumatic brain injury (*n* = 68), the highest AUC to predict brain hypoperfusion was observed for the combination of ICP, PbtO_2_ at baseline and OxR (0.79 [0.69–0.90]), which was significantly better than ICP alone (*p* = 0.01) but not significantly different than ICP and PbtO_2_ or ICP and OxR (Additional file 1: Table S5).

## Discussion

In this retrospective observational study, we observed that a multimodal evaluation including ICP and PbtO_2_ could more accurately detect brain hypoperfusion in a heterogeneous population of brain-injured patients. The dynamic hyperoxia test, which allowed to compute the OxR, did not significantly improve the diagnostic accuracy of this multimodal approach to detect brain hypoperfusion. Similar results were observed when traumatic and non-traumatic brain injuries were analyzed separately.

The most accurate technique to quantify CBF in critically ill patients is CTP; indeed, assessment of CBF velocities using transcranial Doppler cannot provide absolute values of CBF [21], while thermodilution techniques are invasive and require repeated calibrations; their use can be limited in patients with fever or when the probe is placed close to large vessels and suffer from progressive drift of measured CBF values, which can result in inappropriate therapeutic decisions [22]. As CTP required patient’s transportation (i.e., increased risk of hypotension, hypoxemia, increased ICP) and is associated with not neglectable radiations exposure [12], bedside surrogates of CBF are necessary to provide continuous and reliable assessment of brain hemodynamics in acute brain-injured patients. Elevated ICP is often used in clinical practice to identify patients at risk of brain hypoperfusion; however, CBF can also be within high ranges after TBI, indicating hyperemia, which would result in a poor correlation of ICP with absolute CBF values [23]. Moreover, brain hypoperfusion can occur also in brain-injured patients with ICP values below the cutoff of 20–22 mmHg, which has been commonly used to define “intracranial hypertension”, independently from cerebral perfusion pressure (CPP) values [14]. Our data are in line with previous studies that reported a limited accuracy for ICP and CPP to predict CBF values or brain hypoperfusion, while a good correlation between PbtO_2_ and CBF was observed [24–26]; moreover, we showed that this correlation was present also for non-traumatic brain injuries, such SAH and ICH. Importantly, the PbtO_2_ probe was placed into the “at-risk” area (i.e., normal appearing but close to a contusion or injured region), and our results might not be applicable in cases where probe insertion might target different cerebral areas. Moreover, we focused only on the area surrounding the PbtO_2_ catheter, as we included not only patients with diffuse brain injury (i.e., as it might be the case for TBI patients with diffuse axonal injury), but also many with focal injury (i.e., traumatic contusion or intracerebral hemorrhage), in whom the regional CBF might not correlate adequately with the global CBF of the ipsilateral cerebral hemisphere.

Importantly, median ICP and PbtO_2_ were within normal values, i.e., clinical scenarios where performing additional measures, including neuroimaging, can be debatable. As such, our findings should be considered as physiological investigations of the relationship between CBF and neuromonitoring data rather than a support to perform more frequently CTP in this setting.

Previous studies have tried to improve the accuracy of multimodal neuromonitoring to detect brain hypoperfusion by adding, as an example, cerebral microdialysis (i.e., in particular, reduced cerebral glucose or high lactate to pyruvate ratio, which might suggest anaerobic metabolism occurring because of tissue hypoxia) [15]. However, cerebral microdialysis is available only in few centers, and interpretation of its data requires one-hour fluid collection, i.e., it might not be sensitive enough to rapid changes in CBF, which could be detected by monitoring systems providing real-time values. The dynamic hyperoxia test at the bedside could potentially help to improve the accuracy of multimodal neuromonitoring to detect brain hypoperfusion. Indeed, normal PbtO_2_ might be still associated with brain hypoperfusion in patient treated with permissive hyperoxia (i.e., PaO_2_ > 150 mmHg) [27]; in this setting, normal PbtO_2_ values would not reflect normal CBF values but the high levels of dissolved oxygen at the arterial capillary side, which might increase interstitial oxygen diffusion and global delivery. Moreover, low PbtO_2_ values could be observed in the presence of normal or high CBF values, in particular in case of reduced arterial oxygen content (i.e., anemia or hypoxemia) or increased cerebral oxygen consumption (i.e., fever, agitation or fever) [16]. In one study, the OxR was weakly but significantly correlated with ICP and CPP in TBI patients [17]; no direct CBF assessment was performed in this population. However, in a subgroup of patients in whom hyperventilation (i.e., inducing a reduction of CBF) was performed, the OxR was significantly reduced by more than 10%, suggesting a potential relationship between the magnitude of PbtO_2_ response to hyperoxia and the baseline CBF. In another study including 83 TBI patients, the OxR was significantly different across different ranges of CBF values, being lower for CBF of < 10 or 11–15 mL/100 g × min and higher for CBF > 40 mL/100 g × min) [28].

Which are the clinical implications of our findings? As the increase in PbtO_2_ following hyperoxia might be reduced in the presence of low CBF, the OxR might be easily used at the bedside to identify patients at risk of brain hypoperfusion. Although absolute OxR values did not increase the accuracy of ICP and baseline PbtO_2_ to detect brain hypoperfusion in our cohort, the presence of low OxR values (i.e., < 0.2) could identify still some patients with low CBF values despite ICP and PbtO_2_ within “normal ranges”. Conversely, normal OxR in the presence of slightly elevated ICP with still normal PbtO_2_ might suggest the presence of cerebral hyperemia; also, isolated low PbtO_2_ with normal OxR might imply an imbalance between oxygen delivery and consumption that is independent from CBF, i.e., low oxygen content or increased oxygen consumption. This might help to further individualize patients’ care according to the underlying mechanisms resulting in tissue hypoxia. Importantly, it is important to consider that CTP, especially if used in isolation, had limited diagnostic utility to predicting infarct after ischemic stroke [29]; hence, using one single CTP imaging as the “gold standard” to assess hypoperfusion can be somewhat debatable, and, although being used in other studies [15], will deserve further confirmatory analyses in brain-injured patients.

This study has several limitations to acknowledge. First, the study was single-center and local practices might limit generalizability of the results. Second, we included both traumatic and non-traumatic injuries; although these diseases have a significant heterogeneity in pathophysiology and overall management, main results about OxR were similar in the subgroup analysis. Third, we did not specifically assess whether OxR, on admission or repeatedly measured during the ICU stay, might be associated with patients’ outcome, as suggested into another study [17]. Also, we did not evaluate whether fluctuations of ICP and PbtO_2_ might also provide more clinically relevant information on brain perfusion than baseline ICP/PbtO_2_ values or OxR in this setting. Fourth, we did not assess how CBF might respond to hyperoxia; in previous studies, the authors observed a slight decrease in ICP following breathing FiO_2_ 100%, which might suggest intact autoregulatory mechanisms, resulting in vasoconstriction in response to elevated oxygen pressure to maintain a constant tissue oxygen delivery [28]. Fifth, the prevalence of brain hypoperfusion in our study was particularly high, reflecting clinical decision of the attending physicians to explore patients at risk of brain hypoperfusion. A prospective study including all brain-injured patients with neuromonitoring, independently on the pretest probability of brain hypoperfusion, could provide a more extensive and reliable assessment of the role of OxR to detect low CBF values in this setting. Sixth, PbtO_2_ probes were placed in the anterior and middle cerebral artery territories; therefore, changes in posterior vascular territories were not specifically evaluated. Seventh, one may argue that, in the small area where brain oxygenation is measured, CBF would be highly negatively impacted by the instrumented probe. However, regional CBF varied across a wide ranges of values in our study, i.e., many patients had normal or high CBF values. If the tip would have been a reason for low CBF, then oligemia would have been observed in all patients. Moreover, the tip was placed in the region “at risk”, i.e., the cerebral area suffering from contusion, edema or vasospasm; as such, it was expected to have lower regional CBF in the analyzed region than other normal appearing areas (in particular for TBI and ICH). In a previous study [15], a similar methodological approach was used than in our study; also, the authors showed that regional CBF was correlated (although with same variance) with global CBF. Finally, measurement of absolute CBF might not adequately assess the degree of tissue hypoxia; indeed, CBF values within “normal” ranges might still be insufficient for cerebral areas at high metabolic rates or when low arterial oxygen content is present. However, most of therapeutic strategies aim at increasing brain perfusion in acute brain injured patients, and the assessment of CBF remains a relevant end-point in this setting.

## Conclusions

In a heterogeneous population of acute brain-injured patients, cerebral multimodal monitoring with ICP and PbtO_2_ detected regional cerebral hypoperfusion with a higher accuracy than ICP alone. Although the absolute values of OxR, which was derived from a dynamic hyperoxia test, did not significantly increase the accuracy of ICP and PbtO_2_ to detect brain hypoperfusion, low OxR might be still useful to identify those patients with low CBF values despite ICP and PbtO_2_ values within normal ranges.

## Supplementary Information

**Additional file 1: Table S1.** Number of cerebral CT perfusion with brain hypoperfusion (*n* = 56), according to the combination of intracranial hypertension or tissue hypoxia. **Table S2.** Differences in main available data on the day of cerebral CT perfusion (CTP), according to the presence of brain hypoperfusion (i.e., regional cerebral blood flow < 35 mL/100 g × min) in traumatic brain injury (TBI) patients. Data are presented as count (%) or median [IQRs]. **Table S3.** Differences in main available data on the day of cerebral CT perfusion (CTP), according to the presence of brain hypoperfusion (i.e., regional cerebral blood flow < 35 mL/100 g × min) in non-traumatic brain injury (i.e., subarachnoid hemorrhage, SAH or intracerebral hemorrhage, ICH) patients. Data are presented as count (%) or median [IQRs]. **Table S4.** Correlation between regional CBF and monitoring parameters in CTP associated with traumatic brain injury (TBI; *n* = 19). The discriminative ability of each variable or combination to predict cerebral hypoperfusion (i.e., regional CBF < 35 mL/100 g × min) was evaluated using receiver operating characteristic curves with the corresponding area under the curve (AUC), and sensitivity, specificity, positive (PPV) and negative predictive value (NPV) were computed. **Table S5.** Correlation between regional CBF and monitoring parameters in CTP associated with non-traumatic brain injury (*n* = 68). The discriminative ability of each variable or combination to predict cerebral hypoperfusion (i.e., regional CBF < 35 mL/100 g × min) was evaluated using receiver operating characteristic curves with the corresponding area under the curve (AUC), and sensitivity, specificity, positive (PPV) and negative predictive value (NPV) were computed. **Figure S1.** Flowchart of the study. **Figure S2.** Proportion of cerebral CT perfusion with brain hypoperfusion (*n* = 56), according to the combination of two parameters among intracranial hypertension (IH), tissue hypoxia (BH) or low/high oxygen ratio (OxR). **Figure S3.** Proportion of cerebral CT perfusion with brain hypoperfusion, according to the combination of the three parameters, i.e., intracranial hypertension (IH), tissue hypoxia (BH) or low/high oxygen ratio (OxR).

## Data Availability

The datasets used and/or analyzed during the current study are available from the corresponding author on reasonable request.

## Abbreviations

ABG: Arterial blood gas
AUROC: Area under the receiving operator curve
CBF: Cerebral blood flow
CPP: Cerebral perfusion pressure
CTP: Perfusion computed tomography
FO: Favorable neurological outcome
GOS: Glasgow Outcome Scale
ICH: Intracranial hemorrhage
ICP: Intracranial pressure
NPV: Negative predictive value
OxR: Oxygen ratio
PaO_2_: Arterial oxygen pressure
PbtO_2_: Brain tissue oxygen pressure
PPV: Positive predictive value
rCBF: Regional cerebral blood flow
SAH: Subarachnoid hemorrhage
SBI: Secondary brain injury
TBI: Traumatic brain injury
UO: Unfavorable neurological outcome
