# Supplementary material for: Detection of cerebral hypoperfusion with a dynamic hyperoxia test using brain oxygenation pressure monitoring
Source: Crit Care. 2022 Feb 7;26:35. doi: 10.1186/s13054-022-03918-0 (PMC8822803; doi:10.1186/s13054-022-03918-0)

**Supplemental Table 1.** Number of cerebral CT perfusion with brain hypoperfusion (n=56), according to the combination of intracranial hypertension or tissue hypoxia.

|  | Tissue Hypoxia  (n=26) | No Tissue Hypoxia  (n=30) |
| --- | --- | --- |
| Intracranial Hypertension  (n=12) | 5 | 7 |
| No Intracranial Hypertension  (n=44) | 21 | 23 |

**Supplemental Table 2.** Differences in main available data on the day of cerebral CT perfusion (CTP), according to the presence of brain hypoperfusion (i.e. regional cerebral blood flow <35ml/100g x min) in traumatic brain injury (TBI) patients. Data are presented as count (%) or median [IQRs].

|  | ALL  (n=19) | Brain Hypoperfusion (n=14) | No Brain Hypoperfusion (n=5) | p value |
| --- | --- | --- | --- | --- |
| BASELINE | | | | |
| ICP, mmHg | 13 [11-21] | 18 [10-22] | 11 [11-12] | 0.26 |
| CPP, mmHg | 87 [83-97] | 85 [81-94] | 95 [89-108] | 0.09 |
| PbtO_2_, mmHg | 21 [18-25] | 21 [15-22] | 27 [22-28] | 0.07 |
| PaO_2_, mmHg | 107 [80-127] | 101 [79-119] | 107 [101-146] | 0.19 |
| FiO_2_ at baseline, % | 40 [35-48] | 40 [35-50] | 40 [31-40] | 0.62 |
| PaO_2_/FiO_2_ at baseline | 295 [200-368] | 270 [167-357] | 365 [319-370] | 0.13 |
| PEEP, cmH_2_O | 8 [5-8] | 8 [5-10] | 8 [7-8] | 0.89 |
| pH | 7.40 [7.38-7.42] | 7.41 [7.39-7.44] | 7.38 [7.35-7.41] | 0.16 |
| PaCO_2_ baseline, mmHg | 42 [40-47] | 42 [40-46] | 45 [39-47] | 0.82 |
| Sodium, mmol/L | 142 [139-145] | 142 [139-146] | 144 [140-144] | 0.75 |
| Hemoglobin, g/dL | 9.9 [8.5-11.0] | 10 [8.8-11.0] | 9.7 [8.0-10.3] | 0.50 |
| Glucose, mg/dL | 134 [119-145] | 132 [117-144] | 142 [134-163] | 0.34 |
| Body temperature, °C | 37.4 [37.1-37.6] | 37.5 [37.4-37.6] | 36.8 [36.7-37.1] | <0.01 |
| Sedatives, n (%) | 13 (68) | 10 (71) | 3 (60) | 1.00 |
| Opioids, n (%) | 13 (68) | 10 (71) | 3 (60) | 1.00 |
| NMBAs, n (%) | 5 (26) | 3 (21) | 2 (40) | 0.57 |
| Norepinephrine, n (%) | 13 (68) | 9 (64) | 4 (80) | 1.00 |
| Inotropic agents, n (%) | - | - | - | - |
|  |  |  |  |  |
| END OF THE DYNAMIC OXYGEN CHALLENGE | | | | |
| PbtO_2_, mmHg | 67 [52-101] | 65 [41-92] | 123 [96-138] | 0.03 |
| PaO_2_, mmHg | 355 [313-451] | 325 [299-447] | 393 [366-503] | 0.09 |
| pH | 7.39 [7.38-7.44] | 7.39 [7.39-7.47] | 7.40 [7.35-7.42] | 0.56 |
| PaCO_2_, mmHg | 43 [39-46] | 44 [39-46] | 42 [38-48] | 0.89 |
| Oxygen Ratio | 0.23 [0.12-0.29] | 0.21 [0.09-0.27] | 0.31 [0.27-0.37] | 0.09 |
| rCBF, ml/100g x min | 26.4 [14.5-36.1] | 21.3 [13.6-28.3] | 58.5 [45.3-79.3] | <0.01 |

ICP = Intracranial Pressure; CPP = Cerebral Perfusion Pressure; PbtO_2_ = Brain tissue oxygen pressure ; PaO_2_ = arterial blood partial pressure of oxygen; rCBF: regional cerebral blood flow; NMBA = neuromuscular blocking agents

**Supplemental Table 3.** Differences in main available data on the day of cerebral CT perfusion (CTP), according to the presence of brain hypoperfusion (i.e. regional cerebral blood flow <35ml/100g x min) in non-traumatic brain injury (i.e. subarachnoid hemorrhage, SAH or intracerebral hemorrhage, ICH) patients. Data are presented as count (%) or median [IQRs].

|  | ALL  (n=68) | Brain Hypoperfusion (n=42) | No Brain Hypoperfusion (n=26) | P value |
| --- | --- | --- | --- | --- |
| BASELINE | | | | |
| ICP, mmHg | 13 [9-17] | 15 [9-17] | 12 [8-15] | 0.08 |
| CPP, mmHg | 97 [79-113] | 102 [82-116] | 85 [77-104] | 0.11 |
| PbtO_2_, mmHg | 21 [19-23] | 20 [18-21] | 22 [21-24] | <0.01 |
| PaO_2_, mmHg | 111 [99-124] | 116 [99-128] | 110 [98-119] | 0.22 |
| FiO_2_ at baseline, % | 35 [30-40] | 35 [30-40] | 30 [30-35] | 0.08 |
| PaO_2_/FiO_2_ at baseline | 329 [296-364] | 324 [291-356] | 334 [297-367] | 0.42 |
| PEEP, cmH_2_O | 8 [5-10] | 8 [5-10] | 8 [5-10] | 0.59 |
| pH | 7.42 [7.39-7.44] | 7.42 [7.37-7.44] | 7.42 [7.40-7.44] | 0.81 |
| PaCO_2_ baseline, mmHg | 38 [36-41] | 39 [37-41] | 37 [35-39] | 0.06 |
| Sodium, mmol/L | 139 [138-143] | 139 [138-144] | 140 [138-142] | 0.55 |
| Hemoglobin, g/dL | 10.6 [9.5-11.7] | 10.4 [9.8-11.8] | 10.8 [9.0-11.6] | 0.63 |
| Glucose, mg/dL | 134 [122-145] | 134 [119-144] | 136 [126-149] | 0.35 |
| Body temperature, °C | 37.1 [36.8-37.5] | 37.0 [36.5-37.5] | 37.2 [37.0-37.5] | 0.18 |
| Sedatives, n (%) | 35 (51) | 21 (50) | 14 (54) | 0.81 |
| Opioids, n (%) | 41 (60) | 24 (57) | 17 (65) | 0.61 |
| NMBAs, n (%) | 17 (25) | 11 (26) | 6 (23) | 1.00 |
| Norepinephrine, n (%) | 53 (78) | 34 (81) | 19 (73) | 0.55 |
| Inotropic agents, n (%) | 20 (29) | 13 (31) | 7 (27) | 0.79 |
|  |  |  |  |  |
| END OF THE DYNAMIC OXYGEN CHALLENGE | | | | |
| PbtO_2_, mmHg | 79 [56-92] | 62 [49-88] | 90 [81-102] | <0.01 |
| PaO_2_, mmHg | 362 [319-410] | 356 [319-399] | 378 [328-416] | 0.17 |
| pH | 7.42 [7.39-7.43] | 7.42 [7.37-7.43] | 7.42 [7.39-7.43] | 0.94 |
| PaCO_2_, mmHg | 38 [36-40] | 38 [36-40] | 37 [36-40] | 0.91 |
| Oxygen Ratio | 0.23 [0.16-0.29] | 0.20 [0.13-0.27] | 0.28 [0.22-0.33] | <0.01 |
| rCBF, ml/100g x min | 32.4 [24.3-42.9] | 26.2 [17.3-31.5] | 48.9 [39.7-64.0] | <0.01 |

ICP = Intracranial Pressure; CPP = Cerebral Perfusion Pressure; PbtO_2_ = Brain tissue oxygen pressure ; PaO_2_ = arterial blood partial pressure of oxygen; rCBF: regional cerebral blood flow; NMBA = neuromuscular blocking agents

**Supplemental Table 4.** Correlation between regional CBF and monitoring parameters in CTP associated with traumatic brain injury (TBI; n=19). The discriminative ability of each variable or combination to predict cerebral hypoperfusion (i.e. regional CBF < 35 mL/100g x min) was evaluated using receiver operating characteristic curves with the corresponding area under the curve (AUC) and sensitivity, specificity, positive (PPV) and negative predictive value (NPV) were computed.

|  | Correlation with rCBF | AUROC (95% CI) |
| --- | --- | --- |
| ICP | r = -0.27; p=0.13 | 0.69 [0.45-0.92] |
| PbtO_2_ | r = 0.63; p=0.01 | 0.79 [0.57-1.00] |
| Oxygen Ratio | r = 0.56; p=0.04 | 0.77 [0.52-1.00] |
| CPP | r = 0.55; p=0.02 | 0.76 [0.53-1.00] |
| ICP + PbtO_2_ | - | 0.84 [0.65-1.00] |
| ICP + OxR | - | 0.80 [0.57-1.00] |
| ICP + PbO_2_ + Oxygen Ratio | - | 0.90 [0.75-1.00] |

ICP = Intracranial Pressure; CPP = Cerebral Perfusion Pressure; PbtO_2_ = Brain tissue oxygen pressure

**Supplemental Table 5.** Correlation between regional CBF and monitoring parameters in CTP associated with non-traumatic brain injury (n=68). The discriminative ability of each variable or combination to predict cerebral hypoperfusion (i.e. regional CBF < 35 mL/100g x min) was evaluated using receiver operating characteristic curves with the corresponding area under the curve (AUC) and sensitivity, specificity, positive (PPV) and negative predictive value (NPV) were computed.

|  | Correlation with rCBF | AUROC (95% CI) |
| --- | --- | --- |
| ICP | r = -0.29; p=0.02 | 0.63 [0.49-0.76] |
| PbtO_2_ | r = 0.36; p<0.01 | 0.74 [0.61-0.86] |
| Oxygen Ratio | r = 0.57; p<0.01 | 0.74 [0.62-0.86] |
| CPP | r = -0.10; p=0.41 | 0.38 [0.25-0.52] |
| ICP + PbtO_2_ | - | 0.77 [0.66-0.88] |
| ICP + OxR | - | 0.77 [0.65-0.88] |
| ICP + PbO_2_ + Oxygen Ratio | - | 0.79 [0.69-0.90] |

ICP = Intracranial Pressure; CPP = Cerebral Perfusion Pressure; PbtO_2_ = Brain tissue oxygen pressure

**Supplemental Figure 1.** Flow-chart of the study.


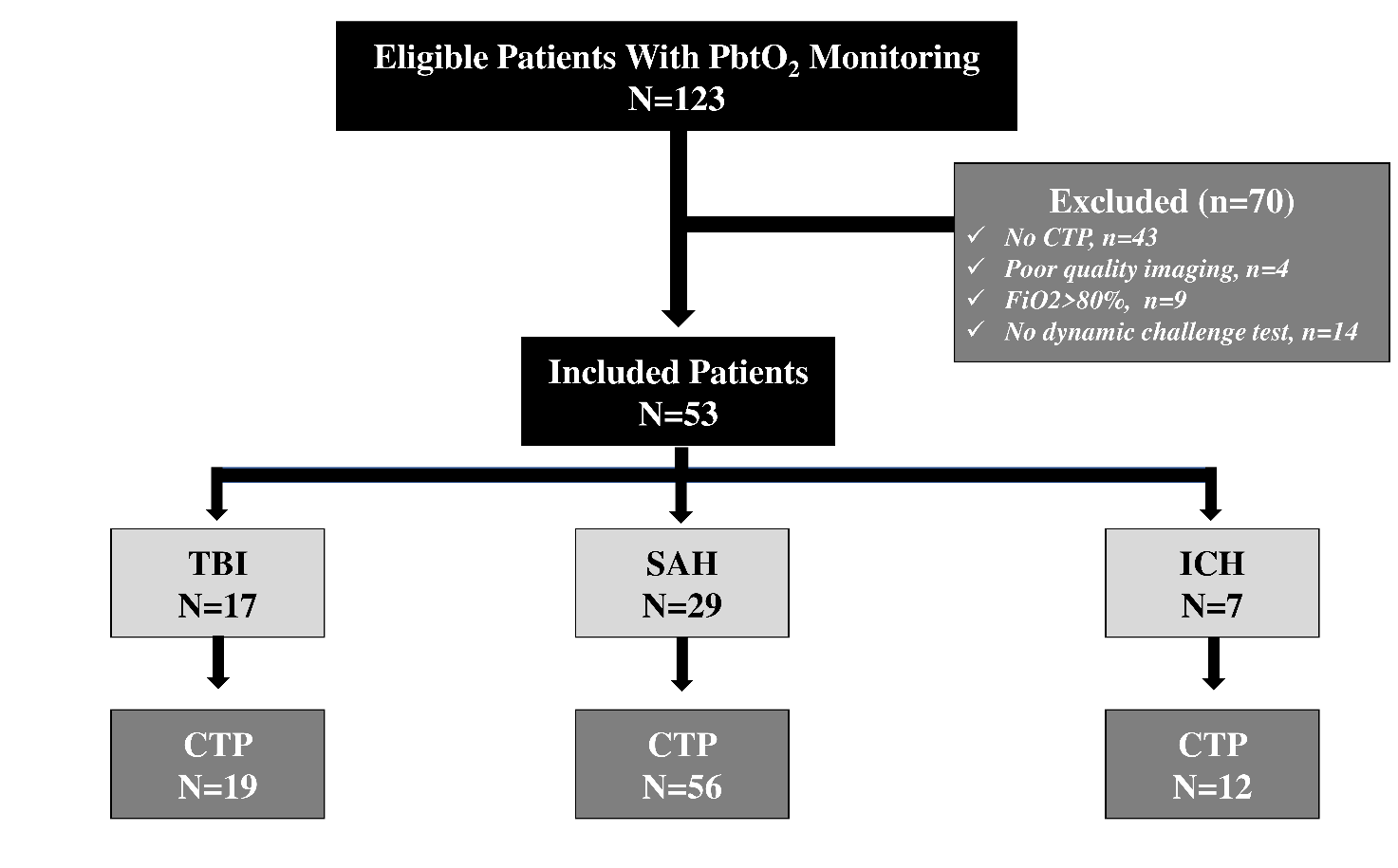


PbtO_2_ = brain oxygen pressure; CTP = Perfusion CT-scan; TBI = traumatic brain injury; SAH = subarachnoid hemorrhage; ICH = intracranial hemorrhage; FiO_2_ = inspired oxygen fraction

**Supplemental Figure 2.** Proportion of cerebral CT perfusion with brain hypoperfusion (n=56), according to the combination of two parameters among intracranial hypertension (IH), tissue hypoxia (BH) or low/high oxygen ratio (OxR).


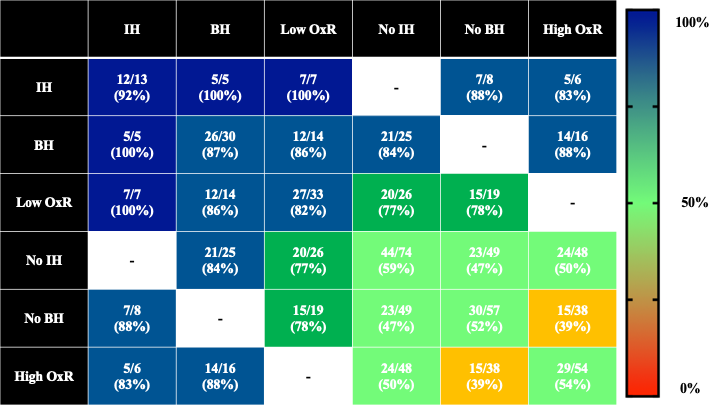


**Supplemental Figure 3.** Proportion of cerebral CT perfusion with brain hypoperfusion, according to the combination of the three parameters, i.e. intracranial hypertension (IH), tissue hypoxia (BH) or low/high oxygen ratio (OxR).


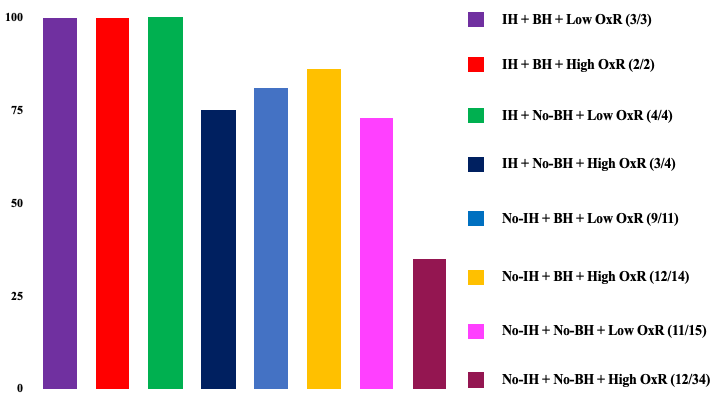

Supplement: Supplementary file 1 — Additional file 1: Table S1. Number of cerebral CT perfusion with brain hypoperfusion (n = 56), according to the combination of intracranial hypertension or tissue hypoxia. Table S2. Differences in main available data on the day of cerebral CT perfusion (CTP), according to the presence of brain hypoperfusion (i.e., regional cerebral blood flow < 35 mL/100 g × min) in traumatic brain injury (TBI) patients. Data are presented as count (%) or median [IQRs]. Table S3. Differences in main available data on the day of cerebral CT perfusion (CTP), according to the presence of brain hypoperfusion (i.e., regional cerebral blood flow < 35 mL/100 g × min) in non-traumatic brain injury (i.e., subarachnoid hemorrhage, SAH or intracerebral hemorrhage, ICH) patients. Data are presented as count (%) or median [IQRs]. Table S4. Correlation between regional CBF and monitoring parameters in CTP associated with traumatic brain injury (TBI; n = 19). The discriminative ability of each variable or combination to predict cerebral hypoperfusion (i.e., regional CBF < 35 mL/100 g × min) was evaluated using receiver operating characteristic curves with the corresponding area under the curve (AUC), and sensitivity, specificity, positive (PPV) and negative predictive value (NPV) were computed. Table S5. Correlation between regional CBF and monitoring parameters in CTP associated with non-traumatic brain injury (n = 68). The discriminative ability of each variable or combination to predict cerebral hypoperfusion (i.e., regional CBF < 35 mL/100 g × min) was evaluated using receiver operating characteristic curves with the corresponding area under the curve (AUC), and sensitivity, specificity, positive (PPV) and negative predictive value (NPV) were computed. Figure S1. Flowchart of the study. Figure S2. Proportion of cerebral CT perfusion with brain hypoperfusion (n = 56), according to the combination of two parameters among intracranial hypertension (IH), tissue hypoxia (BH) or low/high oxygen rat [file 13054_2022_3918_MOESM1_ESM.docx]
